# Supplementary material for: Glucose restriction drives spatial reorganization of mevalonate metabolism
Source: eLife. 2021 Apr 7;10:e62591. doi: 10.7554/eLife.62591 (PMC8057812; doi:10.7554/eLife.62591)
Supplement: Supplementary file 1. [file elife-62591-supp1.docx]

**Supplementary File 1: Table S1.**

Yeast strains used in this study.

| **Strain** | **Description** | **Parent** | **Mat** |
| --- | --- | --- | --- |
| YSR117 | Hmg1-GFP::NAT | W303 | a |
| YSR128 | Hmg1-GFP::NAT *nvj1*Δ | W303 | a |
| YSR136 | Hmg1-GFP::NAT *vac8*Δ | W303 | a |
| YSR12 | Hmg2-GFP::NAT | W303 | a |
| YSR134 | Hmg1_1-525_-GFP::NAT | W303 | a |
| YSR266 | *nvj1*Δ::HYG Hmg1-mRuby3::NAT pRS305::ADH::Nvj1-mNeonGreen::LEU | W303 | a |
| YSR281 | *nvj1*Δ::HYG Hmg1-mRuby3::NAT pRS305::ADH::Nvj1-PX::LEU | W303 | a |
| YSR282 | *nvj1*Δ::NAT Hmg1-mRuby3::HYG pRS305::ADH::Nvj1_Nvj2TM_-mNeonGreen::LEU | W303 | a |
| YSR297 | pRS305::ADH::Nvj1_15-24Δ_-mNeonGreen | W303 | a |
| YSR298 | *nvj1*Δ::NAT Hmg1-mRuby3::HYG pRS305::ADH::Nvj1_15-30Δ_-mNeonGreen | W303 | a |
| YSR214 | Hmg1-GFP::NAT *upc2*Δ::HYG | W303 | a |
| YSR148 | *nvj1*Δ::HYG | W303 | a |
|  | *tgl3,4,5*Δ | W303 | a |
| YSR176 | Hmg1-GFP::NAT *osh1*Δ | W303 | a |
| YSR193 | Hmg1-GFP::NAT *ltc1*Δ | W303 | a |
|  | Hmg1-GFP::NAT Nvj1-mRuby3::HYG | W303 | a |
| YSR149 | Hmg1-GFP::NAT *snf1*Δ::HYG | W303 | a |
| YSR213 | Hmg1-GFP::NAT *ecm22*Δ::HYG | W303 | a |
| YSR167 | Hmg1-mRuby3::HYG | W303 | a |
| YSR175 | Hmg1-mRuby3::HYG *nvj1*Δ::NAT | W303 | a |
| YSR296 | Hmg1-DsRed::HYG | W303 | a |
| YSR301 | Hmg1-DsRed::HYG *nvj1*Δ::NAT | W303 | a |
| YSR250 | *hmg1*Δ::HYG *hmg2*Δ::NAT pRS305::ADH::Hmg1-GFP::LEU | W303 | a |
| YSR260 | *hmg1*Δ::HYG *hmg2*Δ::NAT *nvj1*Δ::HIS pRS305::ADH::Hmg1-GFP::LEU | W303 | a |
| YSR183 | Erg10-mNeonGreen::HYG | W303 | a |
| YSR160 | Erg13-mNeonGreen::HYG | W303 | a |
| YSR159 | Erg12-mNeonGreen::HYG | W303 | a |
| YSR187 | Erg8-mNeonGreen::HYG | W303 | a |
| YSR162 | Mvd1-mNeonGreen::HYG | W303 | a |
| YSR161 | Idi1-mNeonGreen::HYG | W303 | a |
| YSR188 | Erg9-mNeonGreen::HYG | W303 | a |
| YSR156 | Erg1-mNeonGreen::HYG | W303 | a |
| YSR186 | Erg7-mNeonGreen::HYG | W303 | a |
| YSR184 | Erg11-mNeonGreen::HYG | W303 | a |
| YSR276 | Ncp1-mNeonGreen::HYG | W303 | a |
| YSR185 | Erg24-mNeonGreen::HYG | W303 | a |
| YSR182 | Erg25-mNeonGreen::HYG | W303 | a |
| YSR251 | Erg26-mNeonGreen::HYG | W303 | a |
| YSR236 | Erg27-mNeonGreen::HYG | W303 | a |
| YSR275 | Erg28-mNeonGreen::HYG | W303 | a |
| YSR270 | Erg6-mNeonGreen::HYG | W303 | a |
| YSR157 | Erg2-mNeonGreen::HYG | W303 | a |
| YSR189 | Erg5-mNeonGreen::HYG | W303 | a |
| YSR158 | Erg4-mNeonGreen::HYG | W303 | a |
| PK1359 | Erg6-mTFP::HIS3 Whi5-mKok::TRP1 Vma1-mNeptune2.5::KAN Nvj1-mRuby3::NAT Msn2-mNeonGreen::HYG | W303 | Diploid |
| PK1261 | Erg6-mTFP::HIS3 Whi5-mKok::TRP1 Vma1-mNeptune2.5::KAN nvj1Δ::NAT  Msn2-mNeonGreen::HYG | W303 | Diploid |
| YSR297 | *nvj1*Δ::NAT Hmg1-mRuby3::HYG pRS305::ADH::Nvj1_RK→AA_-mNeonGreen | W303 | a |
| YSR314 | *nvj1*Δ::NAT *hmg2*Δ::HYG pRS305::ADH::Nvj1-mNeonGreen | W303 | a |
| YSR315 | *nvj1*Δ::NAT *hmg2*Δ::HYG pRS305::ADH::Nvj1_RK→AA_-mNeonGreen |  |  |
| YSR303 | Nvj1-mNeonGreen::HYG | W303 | a |
| YSR304 | NVj1-mNeonGreen::HYG *upc2*Δ::NAT | W303 | a |
| YSR305 | Hmg1-3HA::G418 | W303 | a |
| YSR306 | Hmg1-3HA::G418 *nvj1*Δ::NAT | W303 | a |
