# Supplementary material for: Glucose restriction drives spatial reorganization of mevalonate metabolism
Source: eLife. 2021 Apr 7;10:e62591. doi: 10.7554/eLife.62591 (PMC8057812; doi:10.7554/eLife.62591)
Supplement: Supplementary file 2. [file elife-62591-supp2.docx]

**Supplementary File 2: Table S2.**

Plasmids used in this study.

| **Name** | **Description** | **Purpose** | **Source** |
| --- | --- | --- | --- |
| pSR258 | pRS305::ADH::Nvj1-PX-mNeonGreen::LEU | expression of Nvj1 chimera from ADH promoter | This study |
| pSR280 | pRS305::ADH::Nvj1_Nvj2TM_-mNeonGreen::LEU | expression of Nvj1 TM chimera from ADH promoter | This study |
| pSR294 | pRS305::ADH::Nvj1_15-24Δ_-mNeonGreen::LEU | expression of Nvj1 truncation from ADH promoter | This study |
| pSR295 | pRS305::ADH::Nvj1_15-30Δ_-mNeonGreen::LEU | expression of Nvj1 truncation from ADH promoter | This study |
| pSR233 | pRS305::ADH::Hmg1-GFP::LEU | expression of Hmg1 from ADH promoter | This study |
| pSR242 | pRS305::ADH::Nvj1_RK→AA_-mNeonGreen::LEU | expression of Nvj1 double alanine mutant from ADH promoter | This study |
